# Mechanisms of Digestive Enzyme Response to Acute Salinity Stress in Juvenile Yellowfin Tuna (*Thunnus albacares*)

**DOI:** 10.3390/ani13223454

**Published:** 2023-11-09

**Authors:** Ninglu Zhang, Rui Yang, Zhengyi Fu, Gang Yu, Zhenhua Ma

**Affiliations:** 1 Key Laboratory of Efficient Utilization and Processing of Marine Fishery Resources of Hainan Province, Sanya Tropical Fisheries Research Institute, Sanya 572018, China; ningluz@163.com (N.Z.); janeyhn4321@yeah.net (R.Y.); zhengyifu@163.com (Z.F.); gyu0928@163.com (G.Y.); 2 South China Sea Fisheries Research Institute, Chinese Academy of Fishery Sciences, Guangzhou 510300, China; 3 College of Science and Engineering, Flinders University, Adelaide 5001, Australia

**Keywords:** fish, gut, acute hyposalinity stress, digestibility, intestinal morphology

## Abstract

**Simple Summary:**

Regarding a better understanding of the process of changes in the digestive physiological state of yellowfin tuna (*Thunnus albacares*) and the distribution of digestive enzymes, it will provide data to support common problems during yellowfin tuna culture. For yellowfin tuna, the digestive state affects the measure of yellowfin tuna’s physical health and plays a crucial relationship to its ontogeny in terms of nutrition and immune regulation. However, there are fewer studies on the digestive physiological state of farmed yellowfin tuna in China. In the present study, a control salinity of 32‰ and an experimental salinity of 29‰ in natural seawater were treated for 48 h under abrupt salinity change to identify the digestive enzyme activities in different tissues (stomach, foregut, and pyloric cecum) at different times (0 h, 12 h, 24 h, 48 h). The results of the study will provide data to support the aquaculture process of juvenile yellowfin tuna.

**Abstract:**

This study investigates the effect of a sudden change in salinity for 48 h on the digestive enzyme activity of juvenile yellowfin tuna. The treatment included a control salinity of 32‰ in natural seawater and an experimental salinity of 29‰. Acute stress experiments were carried out on 72 juvenile yellowfin tuna (646.52 ± 66.32 g) for 48 h to determine changes in digestive enzyme activity in different intestinal sections over time (0 h, 12 h, 24 h, 48 h). The activities of pepsin, trypsin, α-amylase, lipase, and chymotrypsin in the digestive organs (stomach, foregut, and pyloric ceca) of juvenile yellowfin tuna were measured. Pepsin and pancreatic protease in the experimental group were significantly lower than in the control group (*p* < 0.05). α-amylase showed a fluctuating trend of decreasing and then increasing, and its activity trend was pyloric ceca > foregut > stomach. The lipase activity of gastric tissues decreased at the beginning and then increased, reaching a minimum at 24 h (2.74 ± 1.99 U·g protein^−1^). The change of lipase in the pyloric ceca and foregut was increasing and then decreasing. The lipase activity trend was pyloric ceca > foregut > stomach. The chymotrypsin showed a decreasing and increasing trend and then stabilized at 48 h with a pattern of pyloric ceca > foregut > stomach. Similarly, the gut villi morphology was not significantly altered in the acutely salinity-stressed compared to the non-salinity-stressed. This study suggests that salinity may change the digestive function of juvenile yellowfin tuna, thereby affecting fish feeding, growth, and development. On the contrary, yellowfin tuna is highly adapted to 29‰ salinity. However, excessive stress may negatively affect digestive enzyme activity and reduce fish digestibility. This study may provide a scientific basis for a coastal aquaculture water environment for yellowfin tuna farming, which may guide the development and cultivation of aquaculture.

## 1. Introduction

*Thunnus albacares* belongs to the order Perciformes, Mackerelidae, Tuna, commonly to referred as yellowfin tuna [1]. Yellowfin tuna is a high-speed swimming fish with pelagic migratory behavior, it is found mainly in the tropical and subtropical waters of the Pacific, Indian, and Atlantic Oceans, and in China is found in the South China Sea, East China Sea and off the coast of Taiwan [2,3]. As an economically valued tuna species, the global annual catch of yellowfin tuna exceeds 1.4 million tons until 2022, making it the second most harvested tuna species in the world, after skipjack tuna (International Seafood Sustainability Foundation 2022). Yellowfin tuna is a fast-growing species of tuna with high flesh quality [4]. It is the species of choice for offshore aquaculture [1,5,6]. Currently, research on yellowfin tuna is focused on biology [7,8], stock and fisheries research [9,10], food nutrient analysis [11,12] evaluation [13,14], and growth and culture. Artificial farming of yellowfin tuna in China is still in its infancy. The Deep Seawater Aquaculture Technology and Species Development Innovation Team of the Chinese Academy of Fisheries Sciences has realized indoor recirculating water and offshore deep-water net tank culture of yellowfin tuna in Lingshui Li Autonomous County, Hainan Province. The team has long been dedicated to research on the culture biology and disease control of yellowfin tuna and has made important progress in indoor recirculating water and offshore deep-water net tank culture [15,16].

Salinity is an important environmental condition for fish life and is one of the most critical factors affecting the physiological activity of fish [17]. Salinity affects the structure, digestive enzyme activity, and physiological function of the corresponding tissues and organs in the fish digestive tract, which in turn affects the digestion and absorption of food, ultimately affecting the growth and development of fish and disrupting their normal physiological and behavioral activities [18,19,20]. Different fish have different abilities and ranges of adaptation to salinity. Generally speaking, fish have the lowest metabolic rates and highest growth rates in environments close to the appropriate salinity for their long-term life. When salinities are too high or too low, fish use more energy for osmotic pressure regulation, resulting in reduced activity of their digestive enzymes, reduced muscle quality, and compromised growth and survival rates. Fluctuations in salinity are also evident in the coastal areas of China. Due to the low-lying topography of China’s coastal areas, they are susceptible to weather extremes, such as typhoons and hurricanes. In turn, these weather extremes may trigger floods and tides, leading to fluctuations in salinity. In addition, river injection may also be a cause of salinity fluctuations which poses a threat to cage and land-based farming that rely on naturally filtered seawater. A low salinity of 28.5‰ has been observed off the coast of Hainan, China [20], but the optimal salinity for yellowfin tuna growth is 31.2 to 33.3‰ [21]. Changes in fish intestinal viability and histomorphology are receiving increasing attention for the long-term purpose of sustainable aquaculture production systems [22]. Limited at this stage to the development of aquaculture-built ecosystem states, fish intestinal enzyme activity and gut status can be inferred from aspects of fish feeding, digestion, energy balance, and health [23], but links to tuna are still lacking. Gut viability and morphology may vary between fish populations [24], so specific information is needed, particularly during changes in the culture environment and fish rearing. 

Pepsin is a digestive protease secreted by the master cell of the gastric mucosa in the stomach. Its function is to break down proteins in food into small peptide fragments. Pepsin is not produced directly by the cell. The master cell secretes pepsinogen, which is stimulated by gastric acid or pepsin to form pepsin [25]. This is a protective mechanism that prevents pepsin from digesting its own proteins within the cells. Trypsin is a serine protein hydrolase and is one of the most widely used proteases available [26,27]. Derived from the hepatopancreas, intestine, and blind pyloric sac of fish, trypsin is the main endogenous protein hydrolase of the fish gut [28], which selectively hydrolyses peptide bonds in proteins made up of the carboxyl groups of lysine or arginine. In vertebrates, it functions as a digestive enzyme [29], which breaks down proteins in food and promotes nutrient absorption. In the pancreas, trypsin is synthesized as precursor trypsinogen and then secreted as a component of pancreatic juice into the duodenum, where it is activated by enterokinase or self-catalysis. Amylase, another indicator of an animal’s digestive capacity, can be divided into α-amylase and β-amylase depending on the configuration of the hydrolysis product. α-amylase can exist in various extreme environments and is often found in the digestive system of aquatic animals [30,31]. Lipases, also known as triacylglycerol acyl hydrolases, catalyze the hydrolysis of triglycerides at the oil-water interface to produce the corresponding fatty acids and glycerol [32,33]. Chymotrypsin is an endoprotease secreted by the pyloric caeca that specifically hydrolyses peptide bonds formed by carboxyl groups of aromatic amino acids or amino acids with large non-polar side chains [34]. 

Temporary environmental fluctuations may pose an unknown challenge to yellowfin tuna larvae in artificial breeding experiments. We aim to study the changes in digestive enzyme activity with salinity during the growth of juvenile tuna and explore the adaptability of the digestive enzyme system of juvenile tuna to salinity, to accumulate experimental data and research materials for the breeding, ecophysiology, and domestication of yellowfin tuna due to low salinity water.

## 2. Materials and Methods

### 2.1. Experimental Methods and Design

**Figure 1:**
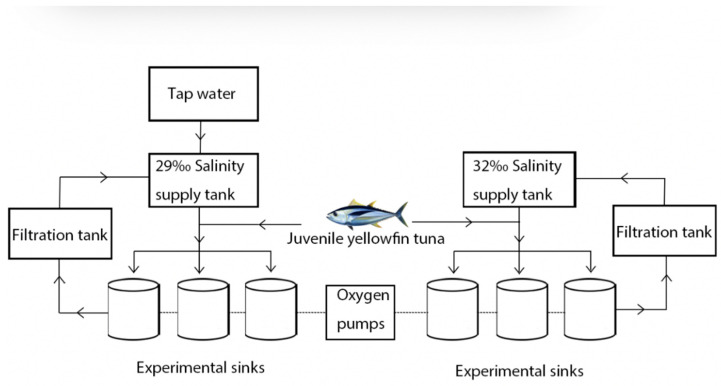
Experimental design of acute hyposalinity on digestive enzyme activity and intestinal tissue sectioning in juvenile yellowfin tuna (*Thunnus albacares*).

The Lingshui Research Station provided the juvenile yellowfin tuna, Tropical Aquatic Research and Center, South China Sea Fisheries Research Institute, Chinese Academy of Fisheries Sciences, China. The body length is 28.97 ± 2.17 cm, and the body weight is 646.52 ± 66.32 g. Seventy-two juvenile yellowfin tuna were randomly placed in six 5000 L fiberglass tanks equipped with a recirculating filtered seawater system for a 7-day period of domestication. Fresh miscellaneous fish pieces (4 cm × 2 cm) were fed daily from 08:30 to 09:00 at 5–8% of body weight per day. No feeding was provided the day before and during the experiment. The experimental installation was divided into a fiberglass water tank, a supply tank, a filtration basin, a circulating water control system, and an oxygen pump. To maintain the water temperature, salinity, and light control, automatic water changes were carried out through the electrical control system. During the experiment, each fiberglass tank was supplied with filtered seawater at a water exchange rate of 300% of the daily tank capacity. Each experiment was replicated three times with 32‰ as the control group and 29‰ as the pressure group (Figure 1). 

The salinity of the stress group was adjusted gradually, at a rate of 1‰ to prevent instability of salinity caused by a rapid rate therefore, naturally filtered seawater was added to tap water that had been aerated for 24 h. The experiment was initiated when the salinity of the water in the stress group reached 29‰. The experiment was carried out over a period of 48 h. Illumination time was maintained at 14:10 h (light:dark). Salinity, water temperature, dissolved oxygen (DO), and pH were monitored using the HQ40d multi-parameter instrument (HQ40d18, Hach, Loveland, CO, USA), and NaNO_2_ and NH_3_-N were monitored using the Chinese biotechnology (Zhecheng Biotechnology, Beijing, China). During the experiment, the temperature of the water is maintained at 29.5 ± 0.5 °C, DO > 7.50 mg·L^−1^, pH 7.93 ± 0.12, NaNO_2_ < 0.1 mg·L^−1^ and NH_3_-N < 0.05 mg·L^−1^.

### 2.2. Analytical Method

Samples were collected from each experimental group at 0 h, 6 h, 24 h, and 48 h of the experiment. Three juvenile yellowfin tuna were randomly collected from the experimental and control groups at each time point for digestive enzyme activity assay and histological analysis. Fish were anesthetized using 200 mg·L^−1^ MS-222 and dissected on ice trays. The stomach, pyloric ceca, and foregut samples were quickly removed, rinsed with pre-cooled saline, blotted on filter paper, placed in frozen centrifuge tubes, and stored at −80 °C. Samples were partially removed prior to the assay, thawed in a refrigerator at 0–4 °C, 0.1–0.2 g of the tissue sample were weighed, then pre-chilled saline was added at 9 times the volume and homogenized using a glass homogenizer on ice with 0.2 M NaCl and centrifuged (0–4 °C, 2500 r·min^−1^, 10 min). Incubate supernatant in enzyme substrate and the digested enzyme activity was read on a spectrophotometer (Synergy H1, BioTek Instruments, Winooski, VT, USA). The triplicate method is used for each data item. Juvenile yellowfin tuna foregut was collected and fixed in 4% paraformaldehyde (500 ML, BL 539A, Biosharp, Hefei, China). The fixed tissue was embedded in paraffin blocks using a Leica RM 2016 rotary slicer (Shanghai Leica Instrument Co., Ltd., Shanghai, China) and cut into a series of cross sections (4 µm thick). A histological analysis was carried out using hematoxylin-eosin (HE) staining. Each slide with tissue sections was permanently fixed with a neutral ball. Sections were observed, photographed, and preserved using an inverted biomicroscope (DMI8, Leica, Wetzlar, Germany).

A protein quantification kit (catalog No: A045-4, built in Nanjing, China) was used to determine the protein content by the Thomas Brilliant Blue method using bovine serum protein as a standard. The protein concentration by the microplate colorimetric method was incubated at 37 °C for 30 min at 562 nm. The Pepsin Assay Kit (Catalogue No: A080-1-1, built in Nanjing, China) was used to determine the amount of pepsin in gastric tissue. Pepsin hydrolyzes protein to produce phenol-containing amino acids, and the phenol-containing amino acids reduce the phenol reagent to a blue substance by colorimetric incubation at 37 °C for 20 min at 660 nm and the absorbance is measured to calculate its activity. An activity unit is defined as 1 µg of tyrosine per mg of histone at 37 °C, enzymatic minute of proteolysis produces 1 µg of tyrosine which is equivalent to 1 enzyme activity unit (1 enzyme activity unit = 1 µg of tyrosine·min·mg^−1^ of histone). Trypsin activity in tissues was determined with a kit (Catalogue No: A080-2, built in Nanjing, China). Trypsin catalyzes the hydrolysis of the ester chain of the substrate ethyl arginate, causing an increase in its absorbance value at 253 nm, and the enzyme activity can be calculated from the change in absorbance. By colorimetric method, the absorbance values were measured, and the activity was calculated by adjusting distilled water to zero using a UV spectrophotometer at 253 nm using a 0.5 cm quartz cuvette. A unit of enzyme activity is defined as a change in absorbance of 0.003 per minute of trypsin per mg of protein at pH 8.0, 37 °C. The α-amylase Assay Kit (Catalogue No: C016-1-1, built in Nanjing, China) was used to determine the activity of α-amylase in fish visceral tissues. α-amylase hydrolyses starch to produce glucose, maltose, and dextrin. The amount of starch hydrolyzed can be deduced from the shade of blue, and the activity of α-amylase can be calculated by adding iodine solution to the unhydrolyzed starch to produce a blue complex if the substrate concentration is known and in excess. The absorbance values were measured, and the activity was calculated by a colorimetric method using a 1 cm optical diameter colorimetric cup adjusted to zero with distilled water at 660 nm. The unit of activity was 1 unit of amylase activity equal to 1 mg of protein in the tissue hydrolyzing 10 mg of starch at 37 °C for 30 min with the substrate. The Lipase Assay Kit (Catalogue No: A054-1-1, built in Nanjing, China) was used to determine the lipase activity in fish visceral tissues. The emulsion made from triglycerides and water has an emulsified nature due to the absorption and scattering of incident light by its micelles. The triglycerides in the micelles are hydrolyzed by the action of lipase, causing the micelles to split and the scattered light or turbidity to be reduced. As a result, the rate of reduction is related to the lipase activity. This was determined by a colorimetric method in which a spectrophotometer is metered at 420 nm, a 1 cm optical diameter glass cuvette zeroed with the Tris buffer allowed the uptake of the tissue to be measured and its viability was calculated. The unit of activity is one unit of enzyme activity for each 1 µmol of substrate consumed per gram of histone that reacted with the substrate in this reaction system for 1 min at 37 °C. The Chymotrypsin Assay Kit (Catalogue No: A080-3-1, built in Nanjing, China) was used for the determination of chymotrypsin activity in animal tissues. The chymotrypsin assay uses casein as a substrate. Chymotrypsin hydrolyses the protein to produce phenol-containing amino acids, and the phenol reagent is reduced to a blue substance by the phenol-containing amino acids. The chymotrypsin activity can be determined using colorimetry. Incubate for 20 min at 37 °C, 660 nm, with a 1 cm optical diameter cuvette, zeroed with distilled water, colorimetric, and calculate the activity. Activity units are equivalent to 1 enzyme activity unit per mg of histone protein per minute of protein breakdown at 37 °C to produce 1 µg of amino acids.

### 2.3. Statistical Analysis

SPSS 26.0 statistical software was used for statistical analysis of all data, and differences in enzyme activity between groups were compared between control and stress groups using a two-way ANOVA. Differences were considered significant when *p* < 0.05 and not significant when *p* > 0.05. Plots were made using Origin software (2019 edition). Data results were expressed as mean ± standard deviation (mean ± SD). 

## 3. Results

### 3.1. Effect of Salinity Changes on Pepsin and Trypsin Activity

**Figure 2:**
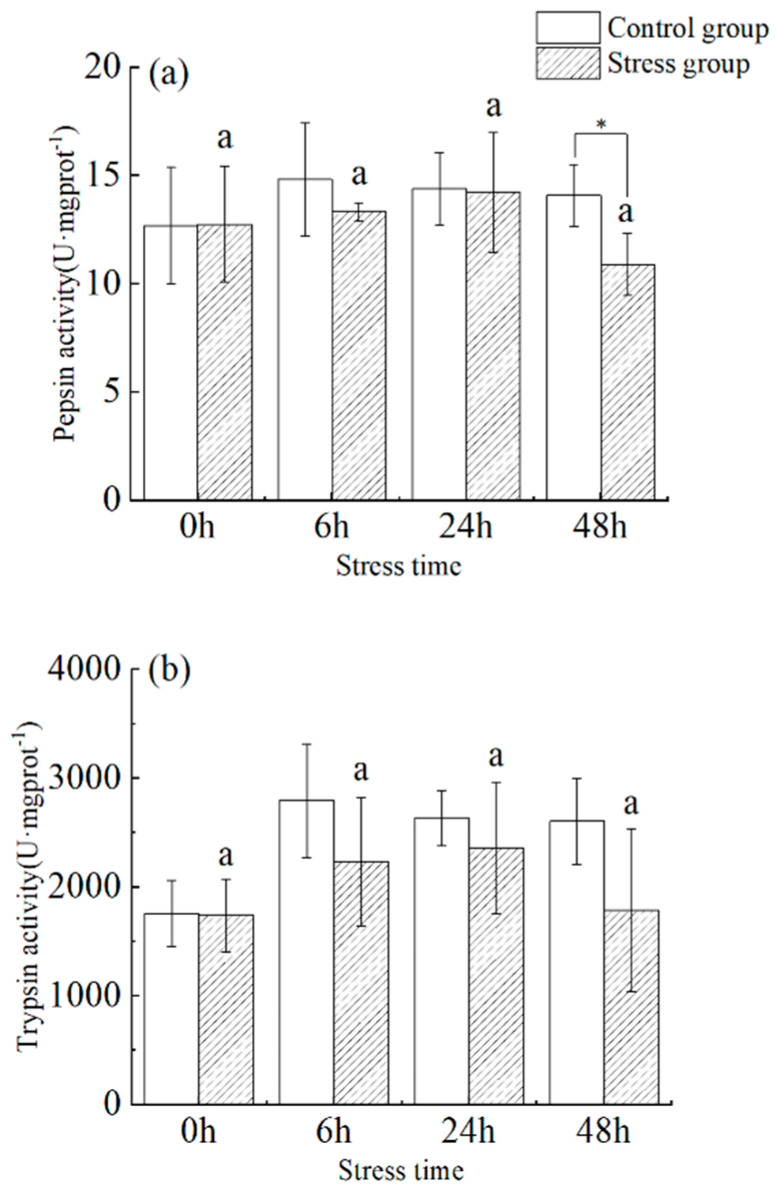
Effect of acute hyposalinity stress on pepsin and trypsin activity in juvenile yellowfin tuna (*Thunnus albacares*), (*n* = 9). (**a**) Pepsin activity, (**b**) trypsin activity. At the same salinity, different letters at different time points indicate a significant difference (*p* < 0.05). Different letters indicate differences in experimental groups at different times, and * indicates differences between the experimental or control groups.

Under stress, pepsin activity increased with time from 0 h to 48 h (Figure 2a). There was no significant difference between 0 h and 48 h (*p* > 0.05), and the maximum value occurred at 24 h (14.208 ± 2.774 U·mg protein^−1^), followed by a decrease to a minimum value (10.887 ± 1.440 U·mg protein^−1^) at the 48 h. Pepsin activity in fish from the stress group was significantly lower than that of the control group at 6 and 48 h (*p* < 0.05). 

In the stress group, there was a slight fluctuation in trypsin activity in the pylorus of juvenile yellowfin tuna between 0 h to 48 h as the duration of stress increased (Figure 2b), showing a trend of increasing (35.8%) and then decreasing (−24.3%), reaching a maximum value (2357.97 ± 602.61 U·mg protein^−1^) at 24 h. There was no significant difference within groups between sampling times and within the same sampling time. In addition, there was no significant difference between different sampling times, versus within the same sampling period, between the control and stress groups (*p* > 0.05).

### 3.2. Effect of Salinity Changes on α-Amylase Activity

**Figure 3:**
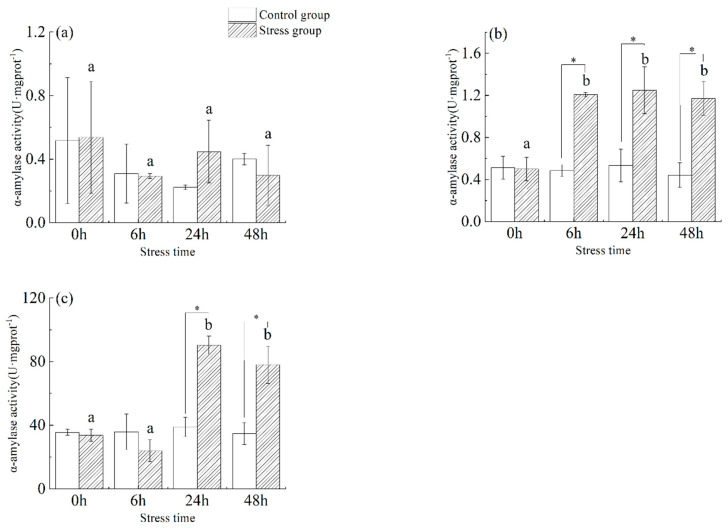
Effect of acute hyposalinity stress on α-amylase activity in juvenile yellowfin tuna (*Thunnus albacares*), (*n* = 9). α-Amylase activity values of yellowfin tuna stomach tissue (**a**), foregut tissue (**b**), and pyloric ceca tissue (**c**). At the same salinity, different letters at different time points indicate a significant difference (*p* < 0.05). Different letters indicate differences in experimental groups at different times, and * indicates differences between the experimental or control groups.

As shown in Figure 3, in the stomach tissue (Figure 3a) of juvenile yellowfin tuna, there was a slight change in α−amylase activity in the stress group from 0 h to 48 h with increasing stress time, but there was no significant difference between the control and stress groups at different sampling times and the same sampling time (*p* > 0.05). In the foregut tissue (Figure 3b) of juvenile yellowfin tuna, α−amylase activity was significantly higher in the stress group from 6 h to 48 h than in the control group (*p* < 0.05) and was not significantly different (*p* > 0.05), reaching a maximum at 24 h (1.25 ± 0.22 U·mg protein^−1^). In the pyloric ceca (Figure 3c) tissue of juvenile yellowfin tuna, the α-amylase activity of the stress group was significantly higher than that of the control group from 24 h to 48 h (*p* < 0.05), and the α-amylase activity of the stress group showed a trend of decreasing and then increasing compared with that of the control group. After 48 h of acute salinity stress, the α-amylase activity of each digestive organ was ranked as follows: pyloric ceca > foregut > stomach.

### 3.3. Effect of Salinity Changes on Lipase Activity

**Figure 4:**
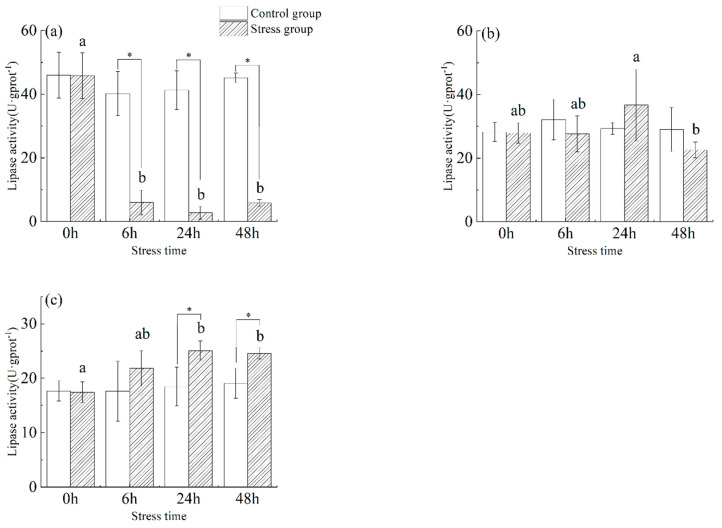
Effect of acute hyposalinity stress on lipase activity in juvenile yellowfin tuna (*Thunnus albacares*), (*n* = 9). Lipase activity values of yellowfin tuna stomach tissue (**a**), foregut tissue (**b**), and pyloric ceca tissue (**c**). At the same salinity, different letters at different time points indicate a significant difference (*p* < 0.05). Different letters indicate differences in experimental groups at different times, and * indicates differences between the experimental or control groups.

As shown in Figure 4, the lipase activity in the stomach (Figure 4a) of juvenile yellowfin tuna in the stress group was significantly lower than that of the control group from 6 h to 48 h (*p* < 0.05). Gastric tissue lipase activity in the stressed group showed a trend of decreasing (−94.02%) and then increasing (112.47%), reaching a minimum value (2.74 ± 1.99 U·g protein^−1^) at 24 h. In the stress group, lipase activity in the foregut (Figure 4b) first increased by (31.69%) and then decreased (−38.45%), reaching a maximum value (36.70 ± 11.22 U·g protein^−1^) at 24 h and a minimum value (22.59 ± 2.51 U·g protein^−1^) at 48 h. In the pyloric ceca (Figure 4c) of juvenile yellowfin tuna from the stress group, lipase activity was significantly higher (*p* < 0.05) than in the control group from 6 h to 48 h and reached its highest activity at 24 h (25.08 ± 1.76 U·g protein^−1^). After 48 h of acute salinity stress, the lipase activity of each digestive organ was ranked as follows: pyloric ceca > foregut > stomach.

### 3.4. Effect of Salinity Changes on Chymotrypsin Activity

**Figure 5:**
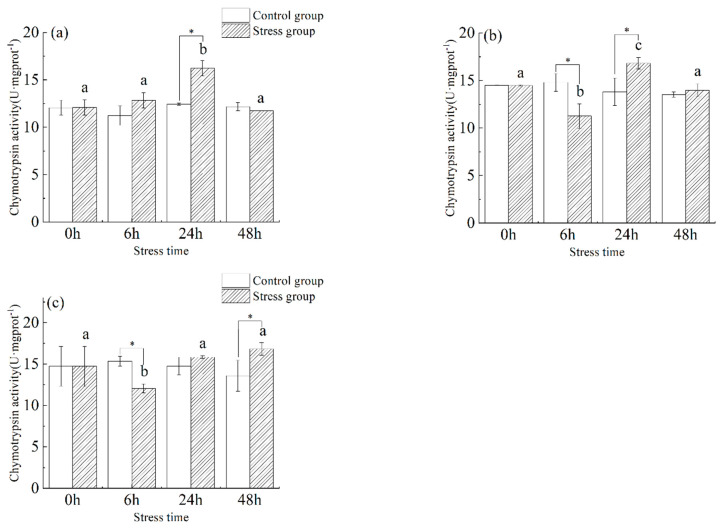
Effect of acute hyposalinity stress on chymotrypsin activity in juvenile yellowfin tuna (*Thunnus albacares*), (*n* = 9). Chymotrypsin activity values of yellowfin tuna stomach tissue (**a**), foregut tissue (**b**), and pyloric ceca tissue (**c**). At the same salinity, different letters at different time points indicate a significant difference (*p* < 0.05). Different letters indicate differences in experimental groups at different times, and * indicates differences between the experimental or control groups.

As shown in Figure 5, the chymotrypsin activity in the stomach (Figure 5a) of juvenile yellowfin tuna showed a trend of increasing (34.3%) and then decreasing (−38.1%), and the chymotrypsin activity in the stomach was significantly higher in the stress group compared to the control group before 24 h (*p* < 0.05) and stabilized at 48 h. The chymotrypsin activity in the stomach reached a maximum at 24 h (16.22 ± 0.81 U·mg protein^−1^) and was significantly higher than in the control group (*p* < 0.05). Chymotrypsin activity in the foregut (Figure 5b) of juvenile yellowfin tuna in the stress group fluctuated slightly from 0 h to 48 h with increasing stress time, with a significant difference between 6 h and 24 h (*p* < 0.05). The chymotrypsin activity in the pyloric ceca (Figure 5c) of juvenile yellowfin tuna experienced a trend of decreasing (−22.4%) and then increasing (39.7%), reaching a minimum value (12.05 ± 0.55 U·mg protein^−1^) at 6 h. The differences in chymotrypsin activity at 24 and 48 h were statistically significant (*p* < 0.05) compared to the control group, the chymotrypsin activity of each digestive organ was ranked as follows: pyloric ceca > foregut > stomach.

### 3.5. Effect of Salinity Changes on Foregut Tissue

**Figure 6:**
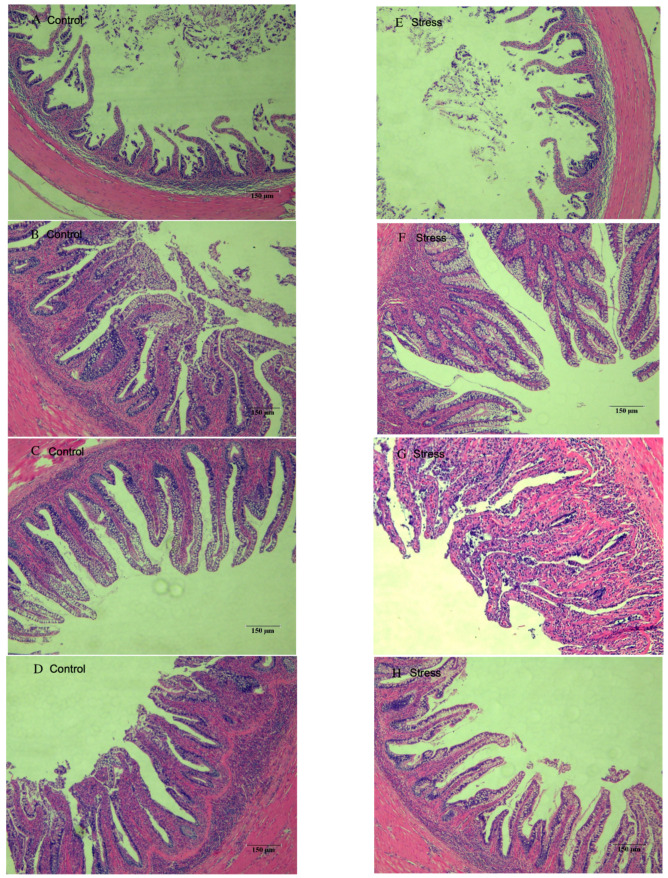
Foregut tissue section of juvenile yellowfin tuna (*Thunnus albacares*) (**A**), Control 0 h; (**B**), Control 6 h; (**C**), Control 24 h; (**D**), Control 48 h; (**E**), Stress 0 h; (**F**), Stress 6 h; (**G**), Stress 24 h; (**H**), Stress 48 h.

Tissue sections of the foregut of each experimental and stress group are shown in Figure 6. The foregut villi were well developed from 6 h–48 h in all experimental groups, and there was no significant difference in villi density. In Figure 6A, the villi appeared sparse, and the intestinal folds were slightly shorter than those in groups B, C, and D, but they were all tightly arranged and morphologically intact. The foregut in the stress group was similar to that of the experimental group, with a few villi having blurred margins and being more closely arranged.

## 4. Discussion

Digestive enzymes influence nutrient absorption and transformation, and salinity has an important effect on their activity and efficiency [35]. The effect of salinity on the activity of digestive enzymes in fish depends on the species, life stage, salinity range, and exposure time of the fish [36]. In general, low salinity generally inhibits the activity of proteases, amylases, and lipases in the digestive tract of fish, affecting the digestion, absorption, and growth of food [37,38]. However, there are some broad-saline or salt-tolerant fish species whose digestive enzyme activity increases with decreasing salinity within a certain low salinity range, showing a strong ability to adapt [39]. It has been shown that changes in salinity within a certain range lead to changes in the activity of digestive enzymes in the digestive tract of fish, which can be broadly classified into three categories: firstly, activation of digestive enzymes [40]. Secondly, the inhibition of digestive enzymes [41], and thirdly, no significant effect on digestive enzymes [42]. In this experiment, with the decrease of salinity on the digestive enzyme activity of the digestive organs of juvenile yellowfin tuna, pepsin, and trypsin showed a decreasing trend, α-amylase, lipase, and chymotrypsin showed an increasing trend except for the stomach tissue which showed a decreasing trend. This proved that the decrease in salinity had an inhibitory effect on the digestive enzyme activity of juvenile yellowfin tuna. Analysis shows that during domestication, when juvenile yellowfin tuna swallow low-salinity seawater, the cells absorb large amounts of water, and the stomach acid becomes overly diluted, resulting in reduced digestibility [43,44,45]. At the same time, the ingestion of large amounts of seawater leads to an increase in inorganic ions in the digestive tract of juvenile yellowfin tuna, many of which are activators or inhibitors of digestive enzymes that can have a direct effect on the enzymes, thus affecting changes in digestive enzyme activity [46,47,48].

For example, Li Xuejiao et al. [49] studied the effects of short-time salinity changes on the blood biochemical parameters and digestive enzyme activity of black sea bream (*Acanthopagrus schlegelii*). Mozanzadeh et al. [50] studied the intestinal protease, amylase, and lipase activity of yellow mackerel (*Acanthopagrus latus*) and Asian sea bass (*Lates calcarifer*) at different salinity gradients for 24 h. Kawai et al. [51] showed some variation in digestive enzyme activity in different digestive organs of fish, which can exhibit some tissue-organ specificity. Our study showed that among the digestive enzymes of juvenile yellowfin tuna, trypsin activity was the highest in α-amylase, lipase, and chymotrypsin activity in pancreatic tissues under the same salinity environment, with obvious tissue organ characteristics. The results of the salmon (*Oncorhynchus keta*) [52] study showed that an appropriate increase in salinity could promote pepsin activity, presumably due to the chloride ion activating the protease. In contrast, in the present study, when juvenile yellowfin tuna were under acute hypersaline stress conditions, the pepsin activity of the stress group was lower than that of the control group by 24 h, and the peptidase activity decreased significantly with time. It is assumed that the activation of pepsin in the stomach of juvenile yellowfin tuna was reduced due to the increased concentration of Na^+^, K^+^, and Cl^−^ plasma. Hieu et al. [37] reported that the trypsin activity of striped catfish (*Pangasianodon hypophthalmus*) did not vary with salinity, which is consistent with the results of this experiment. This indicates that salinity did not affect trypsin in pancreatic tissues. 

Studies in flower eels (*Anguilla marmorata*) and Pacific bicolor eels (*Anguilla bicolor pacifica*) showed a decrease in gastric and intestinal amylase activity with increasing salinity [53]. Contrary to our results some studies have shown that lower concentrations of chloride ions can activate α-amylase activity, while higher concentrations inhibit α-amylase activity [54,55]. Studies on cannabis salmon (*Oncorhynchus keta*) have shown that the direct effect of pH and inorganic ions on enzymes is the main reason salinity affects digestive enzyme activity [56,57]. In our experiment, the amylase activity in the stomach and pyloric ceca of juvenile yellowfin tuna showed a decrease and then an increase. 

The results of the Zhang Longgang et al. [58] study on *Scortum barcoo* show that the inhibition of lipase activity decreases with increasing salinity from 0 to 13, and increases with salinity above 13, with the activation of lipase activity. However, in our study, the stomach tissue of juvenile yellowfin tuna was inhibited by the decrease in salinity and the lipase activity of its foregut and pancreatic tissues showed an increase, which may be due to the different changes in the lipase activity of the digestive system caused by the absence of feeding during the experiment. In the present study, juvenile yellowfin tuna foregut and pancreatic tissues decreased lipase activity with increasing treatment time after 24 h, presumably due to longer treatment times and adaptation to their changing environment. The results of a study on American shad (*Alosa sapidissima*) [38] showed that after treatment with salinities of 0, 7, 14, 21, and 28 g·L^−1^, the specific activity of chymotrypsin was highest in the 21 g·L^−1^ treatment group and did not change significantly in the other treatment groups (7, 14 and 28 g·L^−1^). 

There are not many studies on the effects of chymotrypsin digestion, and from this study, it appears that acute low salt has an agonistic effect on various tissues of juvenile yellowfin tuna [59]. Therefore, a suitable salinity level will increase the activity of digestive enzymes in fish to avoid metabolic disorders in the gastrointestinal tract caused by oxidative stress [38,60]. At the same time, too high or too low salinity has a negative effect on the activity of digestive enzymes and will lead to a reduction in the digestive capacity of fish. Salinity directly affects the enzyme activity of the fish, and enzyme activity directly affects the body functions of the fish [61]. In summary, the pyloric blind sac is more sensitive to acute low-salt stress, followed by the foregut and stomach. Under acute hypersaline conditions, there was a significant effect on digestive enzyme activity in all digestive organs of juvenile yellowfin tuna between time points, but digestive enzyme activity basically stabilized at 48 h in all salinity groups, indicating that juvenile yellowfin tuna have slowly adapted to their environment and have a strong ability to regulate.

The intestinal villi increase the digestive and absorption area of the intestine, making it easier for the nutrients in food to be broken down and absorbed by the digestive juices. The intestinal villi contain cells that secrete various digestive enzymes such as amylase, lipase, and protease. These enzymes play an important role in the absorption of nutrients and in improving growth performance. These enzymes help to break down large molecules such as carbohydrates, fats, and proteins. The presence of intestinal villi are important for the growth and development of the animal, as they improve nutrient utilization and bio-efficiency [62]. The height and width of the intestinal villi are usually regarded as the main indicators to evaluate the functional state of the intestine. The larger the area of the intestinal villi, the larger the absorption area of the intestine and the better the digestion and absorption capacity of the organism [63]. In addition, the intestinal environment influences intestinal villus development, and oxidative stress damage is one of the important factors affecting its development [64]. Salinity directly affects the enzyme activity of the fish, and enzyme activity directly affects the body functions of the fish [65]. This study showed that the morphological structure of the foregut was altered between the control group 0 h and the stress group 0 h, possibly due to the impaired quality of their fillets. Acute hyposalinity had no significant effect on the foregut villi of juvenile yellowfin tuna.

The aim of this paper is to characterize how salinity affects digestive enzyme activity in fish tissues and to focus on the activation and inhibition. There was no significant effect of changes in salinity on digestive enzyme activity based on its previous studies, therefore we worked backward to the fact that digestive enzyme activity affects changes in the digestive organs of the fish body, and thus affects fish feeding, growth, and development. Thus, the focus on aquaculture water bodies is also a focus on their aquaculture industry. Therefore, the moderate reduction of seawater salinity in the culture sea area has basically no effect on its growth and development, which provides basic information for perfecting the domestication and culture of yellowfin tuna under seawater culture conditions.

## 5. Conclusions

In this study, we investigated the effects of acute low salinity on the digestive enzyme activities and intestinal status of juvenile yellowfin tuna (Tetraodontidae), whose digestive capacity could be adapted to a salinity level of 29‰ within 48 h. Under salinity stress, digestive enzyme activity in the fish stomach, foregut, and pyloric ceca resumed to average levels after 48 h. In addition, the tissue quality of the fish foregut was more consistent, indicating that digestive enzyme activity in fish can function normally within this salinity variation to avoid metabolic disturbance in the gastrointestinal tract caused by oxidative stress. In summary, the pyloric ceca are more sensitive to acute low-salt stress, followed by the foregut and stomach. This study contributes to an accurate understanding of the mechanisms affecting digestive physiology under salinity stress conditions. In response to the net-pen culture of yellowfin tuna in harsh environments in the coastal areas of China, the growth and development in an orderly manner improve fish survival and production, thus guiding the development and cultivation of the aquaculture industry.

## Data Availability

The original contributions presented in the study are included in the article. Further inquiries can be directed to the corresponding authors.
